# The Interplay between Autophagy and Redox Signaling in Cardiovascular Diseases

**DOI:** 10.3390/cells11071203

**Published:** 2022-04-02

**Authors:** Barbora Boťanská, Ima Dovinová, Miroslav Barančík

**Affiliations:** Centre of Experimental Medicine, Institute for Heart Research, Slovak Academy of Sciences, Dúbravská Cesta 9, 84104 Bratislava, Slovakia; barbora.botanska@gmail.com (B.B.); ima.dovinova@savba.sk (I.D.)

**Keywords:** Nrf2, redox signaling, autophagy, heart, cardiovascular disease

## Abstract

Reactive oxygen and nitrogen species produced at low levels under normal cellular metabolism act as important signal molecules. However, at increased production, they cause damage associated with oxidative stress, which can lead to the development of many diseases, such as cardiovascular, metabolic, neurodegenerative, diabetes, and cancer. The defense systems used to maintain normal redox homeostasis plays an important role in cellular responses to oxidative stress. The key players here are Nrf2-regulated redox signaling and autophagy. A tight interface has been described between these two processes under stress conditions and their role in oxidative stress-induced diseases progression. In this review, we focus on the role of Nrf2 as a key player in redox regulation in cell response to oxidative stress. We also summarize the current knowledge about the autophagy regulation and the role of redox signaling in this process. In line with the focus of our review, we describe in more detail information about the interplay between Nrf2 and autophagy pathways in myocardium and the role of these processes in cardiovascular disease development.

## 1. Introduction

Important risk factor of cardiovascular disease development is a dysregulation of the balance between pro- and anti-oxidative factors in the organism [1,2,3]. This disbalance is associated with an increased production of reactive oxygen species (ROS) and reactive nitrogen species (RNS). ROS and RNS are constantly produced under normal conditions and play an important role in many signaling pathways involved in the regulation of normal biological functions and physiological processes. Oxidative stress caused by an increased production of free radicals leads to a disruption of redox signaling. In response to pathological conditions associated with oxidative stress, cells develop defense systems to detoxify oxidative species and to maintain normal cell homeostasis. The pathway of redox-sensitive nuclear transcription factor Nrf2 and autophagy both play a key role in orchestrating these processes [4,5,6,7,8,9,10]. There is a close interaction between oxidative stress and autophagy. Changes in the cellular redox state, mediated by the increased production of ROS, involve not only Nrf2-driven antioxidant defense mechanisms but can also induce and regulate autophagy. Moreover, autophagy can be involved in redox metabolism regulation by the elimination of molecules and organelles damaged by oxidative stress [9,11]. On the other hand, autophagy can be regulated via antioxidant cell defense mechanisms and the most relevant interplay between autophagy and oxidative stress response mechanisms is achieved by Nrf2 signaling.

In this review article, we overview the function of autophagy and Nrf2-driven redox signaling in the mechanisms of responses of cardiac cells to pathological conditions with an emphasis on the interrelationships of these two systems. We address the control and regulation of Nrf2 signaling and autophagy pathways under normal and stress conditions. Finally, we summarize the current knowledge about the role and interplay of redox signaling and autophagy in cardiovascular diseases.

## 2. Nrf-2 Redox Signaling in Cell Responses to Oxidative Stress

Reactive oxygen species (ROS) are highly reactive metabolites of molecular oxygen (O_2_). They include hydrogen peroxide (H_2_O_2_), hydroxyl radicals (HO^•^), and superoxide anion radicals (O_2_^•−^). The major source of ROS within cells are mitochondria, and mitochondrial ROS are generated as by-products during oxidative phosphorylation [12]. Other sources or producers of free oxygen radicals are peroxisomes [13], endoplasmatic reticulum [14], and enzymes nicotinamide adenine dinucleotide phosphate (NADPH) oxidase or xanthine oxidase [15]. Reactive nitrogen species (RNS) include peroxynitrite (ONOO^−^) and nitric oxide (NO)). The largest amount of RNS is generated from L-arginin by nitric oxide synthases (NOS) [16]. ROS and RNS are constantly produced under normal conditions and play a role in many signaling pathways involved in the regulation of normal biological and physiological processes [10]. Redox signaling under basal levels of free radicals leads to the maintenance of cellular homeostasis and cell proliferation. A disturbance in the balance between the formation of reactive species and the defense provided by cell antioxidants leads to a disruption in normal redox signaling. The consequence is an excessive production of oxygen and/or nitrogen free radicals which results in oxidative stress [17,18]. Oxidative stress causes damage to important cellular biomolecules (proteins, lipids, and nucleic acid) and in such way contributes to a variety of diseases such as cardiovascular diseases, neurodegenerative disorders, inflammatory diseases, and cancer [1,2,3].

In response to rising levels of reactive free radicals, cells develop a defense antioxidant system to detoxify themselves. This defense system includes several antioxidant enzymes and molecules, and the key role in the regulation of the antioxidant response is played by the Nrf2 (Nuclear factor erythroid 2-related factor 2) signaling pathway. Under normal conditions, the transcription factor Nrf2 is maintained in cytoplasm by its endogenous inhibitor Keap1 (Kelch-like ECH-associated protein 1), where it is constantly degraded by the proteasome via ubiquitination [19]. Furthermore, Keap1 is one of the major sensors of the cellular redox status, acting through binding to Nrf2 and promoting its degradation. Under conditions of excessive ROS production, the cysteine residues of the Keap1 are oxidized. The consequence is a disruption of interaction between Keap1 and Nrf2 and the translocation of free Nrf2 into the nucleus. Importantly, Nrf2 controls the expression of several antioxidant and detoxifying enzymes such as superoxide dismutase (SOD), heme oxygenase 1 (HO-1), catalase (CAT), and NAD(P)H quinone dehydrogenase 1 (NQO1) via binding to antioxidant-response element (ARE) [20,21]. Another mechanisms of Nrf2 regulation involves its phosphorylation by various protein kinase pathways such as mitogen-activated protein kinase cascades (MAPK), glycogen synthase kinase-3 beta (GSK-3β) pathway, Fyn kinase, and protein kinase C (PKC) [22,23,24,25,26,27]. Another signaling pathway, phosphatidylinositol 3-kinase (PI3K/Akt) cascade, is involved in the positive regulation of Nrf2 activity indirectly through the phosphorylation and inhibition of GSK-3β [28].

The role of the Nrf2 signaling pathway in the development of several diseases (cardiovascular, neurodegenerative diseases or cancer) and during aging has been documented [29,30,31,32,33,34,35,36,37]. Furthermore, Nrf2 appears to have either a protective or detrimental effect, depending on its activation stage. In acute phases of activation, Nrf2 drives antioxidant defenses to suppress oxidative stress-mediated cellular dysfunction. On the other hand, the opposite effect occurs upon long-term Nrf2 activation. It has been documented that chronic Nrf2 activation in cardiac-specific transgenic mice leads to hyper-reductive state and hypertrophic cardiomyopathy [38]. An excessive expression of Nrf2 has also been found to mediate the increased development of carcinogenesis and tumor mutations [31,39].

### Nrf-2 Redox Signaling in Myocardium

As is known, Nrf2 plays an important role in maintaining redox homeostasis in cardiac cells and is also a crucial regulatory component of cellular antioxidant defense against oxidative stress in the myocardium (cardiovascular system). Furthermore, Nrf2 can contribute to protecting heart and blood vessels under stress conditions and its activation leads to the prevention and delay of cardiovascular diseases [40]. Several studies have documented the involvement of Nrf2 signaling in mechanisms of cardioprotection and in the regulation of cardiac function at different pathological conditions [41,42,43,44]. An acute activation of Nrf2 was found to play a role in cardioprotection against ischemia/reperfusion injury. Calvert et al. found that cardioprotective effects of hydrogen sulfide against ischemic injury in mice are realized via Nrf2 signaling activation [41]. Another study documented that the intravenous administration of 4-hydroxy-2-nonenal (4-HNE) improved the functional recovery of the left ventricle following ischemia–reperfusion in Langendorff perfused mice hearts and the cardioprotective effects of 4-HNE were associated with Nrf2 activation [44]. Moreover, the cardioprotective effects of 4-HNE were not observed in Nrf2-knockout mice. The positive (protective) role of Nrf2 in myocardial responses to pathological conditions are supported also by several other studies documenting the association of Nrf2 with a higher susceptibility to oxidative stress and increased cardiac injury. Strom and Chen found that the knockout of Nrf2 in mice accelerated their progression to heart failure with a significantly higher mortality rate within 10 days after myocardial infarction induction [42]. It was also documented that mice deficient in Nrf2 have higher myocardial susceptibility to inflammation and oxidative stress [43]. Moreover, in a model of pressure, overload accelerated the loss of functional Nrf2 in heart failure [45]. Global knockout of Nrf2 has also been found to enhance cigarette smoke–induced cardiac dysfunction in mice [46]. Furthermore, Nrf2 has also been found to provide protection against diverse cardiomyopathies associated with oxidative stress [47]. In addition, Nrf2 knockout in mice led to the potentiation of doxorubicin-induced cardiotoxicity and cardiac dysfunction [29]. Another study pointed out the important role of the Akt/GSK-3β/Fyn signaling pathway in the regulation of Nrf2 function in cardiomyopathy [28]. They found that the activation of this intracellular signaling pathway mediated the prevention of cardiomyopathy via Nrf2 upregulation. The contribution of p27(kip1) up-regulation in Nrf2-mediated protection against angiotensin II-induced cardiac hypertrophy were also documented [48]. However, while the acute activation of Nrf2 is cardioprotective [41,44], there is evidence that chronic Nrf2 activation can lead to hypo-reductive stress and may be harmful to cardiac function [38].

## 3. Autophagy

Autophagy is an evolutionary conserved lysosome-dependent-self-repair process which occurs in eukaryotic cells and helps the cell maintain normal homeostasis [9,49,50,51]. A basal level of autophagy is constitutively ongoing in most cell types and is involved in cellular growth and cell metabolism. Autophagy can be further induced under stress conditions, such as nutrient or energy starvation to degrade cytoplasmic material into metabolites that can be used in biosynthetic processes or energy production for cell survival. By the elimination of misfolded proteins, protein aggregates, damaged organelles, or lipid droplets, autophagy protects cells against damage [50,52]. The main autophagy pathways are macro-autophagy, micro-autophagy, and chaperon-mediated autophagy (CMA). These pathways differ in their transport of cytoplasmic components into the lysosomes [7,10,49]. Macro-autophagy and CMA play an important role in the prevention of disease pathogenesis via the degradation of oxidized proteins and protein aggregates, whereas micro-autophagy is mainly involved in organelle remodeling and quality control to maintain cell homeostasis [7,53,54]. Selective mitochondrial autophagy, called mitophagy, plays a specific and important role in cellular responses. This process is responsible for the targeted removal and degradation of dysfunctional mitochondria, and is one of the most important mechanisms of mitochondrial quality control [55,56].

### 3.1. Macro-Autophagy

Macro-autophagy (generally referred as autophagy) is the best studied form of autophagy. This process involves the generation of autophagosome, a double-membrane structure used for the transport of sequestered material towards lysosome. The macro-autophagy process includes the following five distinct steps: (i) initiation, (ii) phagophore nucleation, (iii) autophagosomal formation (elongation), (iv) autophagosome-lysosome fusion, and (v) cargo degradation. Several Atg (AuTophaGy-related) and non-Atg proteins are involved in the regulation of these steps [57].

The regulation of the serine/threonine protein kinase mammalian target of rapamycin (mTOR) is essential in maintaining the normal macro-autophagy function, and the inhibition of mTORC1 is a key process in macro-autophagy initiation [58]. The consequence of this inhibition is Atg13 dephosphorylation, activation of Unc-51-like autophagy activating kinases 1 and 2 (ULK1 and ULK2), and the formation of the ULK1/FIP200/Atg13 complex via an interaction with the focal adhesion kinase family-interacting protein of 200 kDa (FIP-200). This complex plays an important role in the initiation of double-membrane formation. Important regulators of macro-autophagy initiation are AMP-activated protein kinase (AMPK) and the PI3K/Akt pathway, acting through the direct phosphorylation of ULK1 or mTOR inhibition [59,60].

Autophagosomes developed from phagophores during elongation. This step is regulated via two ubiquitination-like conjugation systems, namely ATG5-ATG12 and Atg8/LC3-phosphatidylethanoamine [61,62]. An essential step during elongation is the post-translation modification of microtubule-associated protein light chain 3 (LC3) to LC3-II. The ratio of LC3-II/LC3-I is considered as marker for autophagy. The cleavage of nascent LC3 is achieved by Atg4 and the conversion to LC3-II is continuous through phosphatidylethanolamine (PE). Subsequently, LC3-II is attached to the autophagosomal membrane, which facilitates the fusion of the autophagosomal outer membrane with lysosome to form an autophagolysosome. Finally, the hydrolases in lysosomes degrade the autophagolysosomal contents and the membrane of autophagosomes [63]. LC3-related proteins, γ-aminobutyric-acid-type-A-receptor-associated protein (GABARAP) and Golgi-associated ATPase enhancer of 16 kDa (GATE16) have been reported to have similar roles during autophagy as LC3 [64].

The specific autophagic cargos are ubiquitin-labelled and recognized via autophagy adaptor proteins such as p62, NBR1, NDP52, OPTN, and TAX1BP1 [60,63,64,65]. It was described that adaptor proteins contain a ubiquitin-associated domain (UBA) which interact with the ubiquitin chain on target substrates, and the (LC3)-interacting region (LIR) binds on the lysosomal membrane so as to ensure the degradation of sequestered cargos [65,66,67,68].

### 3.2. Chaperon-Mediated Autophagy

Chaperon-mediated autophagy (CMA) is the most selective form of autophagy. In contrast to other lysosomal degradation pathways, CMA cytosolic proteins with the KFERQ pentapeptide motif are delivered to lysosomes in a molecule-by-molecule fashion instead of through vesicular traffic [69,70,71]. Furthermore, chaperon heat shock 70 kDa protein 8 (HSPA8/Hsc70) plays an important role in the identification of misfolded proteins containing a KFERQ motif. After interaction with target proteins, an Hsc70 complex is formed together with heat shock protein 90 (Hsp90), heat shock protein 40 (Hsp40), and other co-chaperones [54]. This complex binds the lysosome-associated membrane protein-2A (LAMP-2A) which is attached to the lysosomal membrane, and the target protein is then transported into the lysosomal lumen and degraded.

It was suggested that Hsc70 and LAMP2A also participate in chaperon-assisted selective autophagy [72]. During this process, an interaction of the chaperone complex occurs, consisting of BAG3/CHIP/HspB8/Hsp70, with pCHIP ligase being responsible for the interaction of co-chaperons and Bcl-2-associated athanogene (BAG3) with ubiquitinated organelles and their delivery to the ubiquitin-proteasome system. The concomitant recruitment of macro-autophagy receptors, such as SQSTM1/p62 and NBR1, acts as a connecting element between the chaperon-assisted selective autophagy, ubiquitylated protein, and the formation of an autophagosome [72].

CMA is tightly regulated by several factors and protein kinases signaling pathways. Furthermore, mTORC2 activation inhibits CMA and plays an important role here [73]. It was described that the phosphorylation of Akt kinase by mTORC2 and the Akt kinase-mediated phosphorylation of the lysosomal glial fibrillary acidic protein (GFAP) can also lead to an inhibition of CMA. of the importance of Akt kinase in the regulation of CMA is also supported by the fact that the PH Domain and Leucine Rich Repeat Protein Phosphatase 1 (PHLPP1) can stimulate CMA via the dephosphorylation and deactivation of Akt kinase [73]. It was also reported that the inhibition of phosphatidylinositol 3-kinase (PI3K), a regulator of Akt kinase activity, activated CMA by reducing the phosphorylation of GFAP [74]. Additionally, LAMP2A can also be regulated at the transcriptional level by transcription factor Nrf2 and this fact confirms the role of CMA in conditions of oxidative stress [75].

### 3.3. Micro-Autophagy and Mitophagy

Micro-autophagy is a poorly studied form of autophagy. It is known as a pathway which delivers cytoplasmatic substances for degradation directly into lysosomes via the invagination of their membranes [76,77]. The regulation mechanisms of micro-autophagy in mammalian cells are not well understood, but rapamycin has been identified as the first activator of micro-autophagy [78]. Endosomal micro-autophagy is a morphologically related variant of general micro-autophagy. During this process, soluble cytosolic proteins are delivered into late endosomes. It has been documented that selectivity could be provided by Hsc70 [79]. Endosomal micro-autophagy has also been found to degrade the macro-autophagy receptors p62/SQSTM1, NBR1, TAX1BP1 and NDP52 in response to starvation. This may affect the selectivity of macro-autophagy for a better utilization of all cytoplasm components [80].

Mitochondria are cell organelles which are necessary to generate chemical energy and oxygen during oxidative phosphorylation. An impaired mitochondrial function is common in many pathologies, especially in organs with a large number of mitochondria, including brain and heart [81,82]. To prevent the development of pathologies, dysfunctional mitochondria are degraded via selective autophagy, called mitophagy. The removal of dysfunctional mitochondria is essential for maintaining cell health. Mitophagy is mainly regulated via receptor-mediated PTEN-induced kinase 1 (PINK1)/Parkin pathway. This pathway is stimulated by diminished mitochondrial membrane potential (MMP), which causes PINK1 accumulation on the outer mitochondrial membrane (OMM). Then, Pink1 recruits Parkin to activate its E3 ubiquitin ligase activity. Subsequently, Parkin triggers the ubiquitination of OMM proteins and the concomitant activation of autophagy machinery. Autophagy adaptor proteins, such as p62, OPTN, or NDP52 recognize the phosphorylated poly-ubiquitinated chains on mitochondria that drive mitophagy by binding with LC3 on the lysosomal membrane [83,84]. Furthermore, PINK1/Parkin-independent mitophagy is regulated via NIX/BNIP3L, FUNDC1 proteins, which are described as receptors for hypoxia-mediated mitophagy [85,86].

### 3.4. Autophagy in Myocardium

Autophagy is essential for maintaining a normal homeostatic function in cardiac cells via a continual process of removing, repairing, and replacing damaged cellular materials. It also plays an important role in cardiovascular pathologies and several studies have documented autophagy disturbances in many cardiac disease states, including heart failure, cardiac hypertrophy, pressure-overload heart failure, ischemic heart disease, and cardiomyopathies [52,87,88,89,90,91,92,93,94,95]. Increased autophagy during ischemia was found in a mouse model and this increase was considered as a beneficial response leading to the elimination of oxidized and damaged cellular components [96]. Acute cardioprotection associated with autophagy activation was also confirmed during ischemia/reperfusion [97,98]. Moreover, mitophagy activation was found as a potential contributor to protection against ischemia/reperfusion injury, mediated by ischemic preconditioning [99] and the cardioprotection mediated by simvastatin [98]. All of the above mentioned studies showed that autophagy activation plays a role in pro-survival responses in myocardial cells. However, autophagy can have a dual role in the responses of cardiac cells to pathological conditions. It may represent a beneficial adaptive response to stress but can also lead to maladaptive responses which are linked to disease pathogenesis and cell-death induction. It has been found that excessive autophagy may lead to cardiomyocyte death [100]. Moreover, the suppression of excessive autophagy plays an important role in reducing myocardial ischemia/reperfusion injury by hesperidin [101] and the amelioration of autophagy activity was associated with cardiac hypertrophy inhibition [102]. Aliskiren was also found to ameliorate heart hypertrophy by suppressing Ang II-PKCβI-ERK1/2-regulated autophagy [103,104].

The relation between autophagy and cellular pathology in the heart involves not only autophagy modulation (stimulation) at pathological conditions but also a disruption of the heart function by defective myocardial autophagy. It is known that abnormal autophagy can accelerate the occurrence and progression of cardiovascular diseases. For example, the pharmacological suppression of starvation-induced autophagy was identified as a factor leading to heart failure [105]. Mice with cardiomyocyte-specific autophagy deficiency through Atg5 deletion were found to exhibit an impaired regression of cardiac hypertrophy following withdrawal of pressure-overload induced by angiotensin II infusion [106].

## 4. Interplay between Autophagy and Redox Signaling

**Figure 1:**
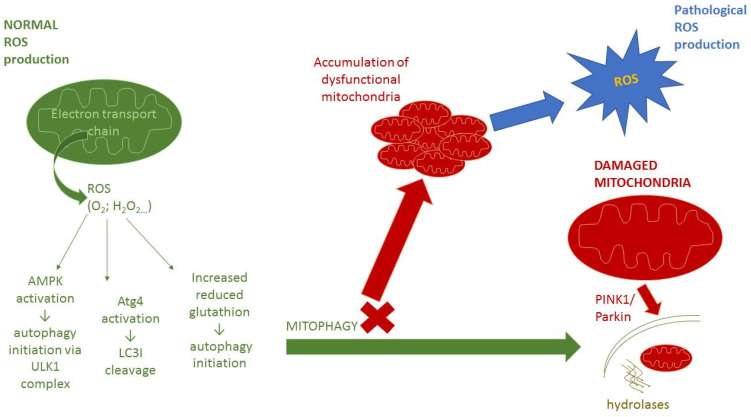
Mitophagy and mitochondrial function. Abbreviations: ROS—reactive oxygen species, O_2_^•−^ superoxide anion radical, H_2_O_2_—hydrogen peroxide, AMPK—AMP-activated protein kinase, ULK1—Unc-51-like autophagy activating kinase, Atg4—AuTophaGy-related protein 4, LC3I—microtubule-associated protein light chain 3-I, PINK1—PTEN-induced kinase ROS are formed in the electron transport chain on the inner membrane of mitochondria. At low levels, ROS are important for normal cell signaling and are also able to positively regulate autophagy through three different mechanisms. At first, activation of AMPK protein kinase via S-glutathionylation of cysteines located in the subunits of AMPK leads to activation of ULK1 complex and subsequent autophagy initiation [111]. Second mechanism involves oxidation of cystein-81 of Atg4, which leads to the cleavage of LC3I to LC3II and facilitates the formation of autophagolysosomes [18]. The last one is the release of reduced glutathione, which can initiate autophagy [112]. These processes contribute to the proper functioning of autophagy/mitophagy and removal of damaged organelles from cells. When the mitophagy process is defective, dysfunctional mitochondria accumulate. The consequence is an increased production of ROS, which can lead to pathological redox signaling and can also disrupt all of these processes.

A close interaction exists between oxidative stress and autophagy [9,10,51]. Changes in the cellular redox state, mediated by increased production of ROS, can both induce and regulate autophagy. On the other hand, autophagy can be involved in the regulation of the redox metabolism by eliminating molecules or organelles damaged by oxidative stress [11]. Mitophagy plays an important role in the elimination of damaged mitochondria, as an important source of ROS (Figure 1). Mitophagy plays a protective role in the quality control of mitochondria and in the restoration of oxidative balance and cellular redox metabolism by reducing free-radical generation [50]. Decreased mitophagy results in ana impaired degradation of damaged and dysfunctional mitochondria, leading to oxidative stress [107]. Mitochondrial dysfunction and increased ROS production is associated with several pathological conditions, including cardiovascular diseases, neurodegeneration, carcinogenesis, and chronic inflammation [108]. This highlights the crucial role of mitophagy in the prevention of cell death and tissue injury. A recent study has described the beneficial effect of mitofusin 2 (MFN2) in reducing angiotensin II-induced cardiomyocytes injury, which was realized via decreased intracellular ROS production in mitophagy regulation [109]. Moreover, the regulation of both mitophagy and redox balance was found to play a role in the ischemic pre-conditioning and post-conditioning induced protection of myocardial function against ischemic injury [110]. Decreased mitophagy, resulting in oxidative stress from dysfunctional mitochondria, may also play a role in mechanisms of premature vascular aging in hypertension [107].

Autophagy can be also regulated via antioxidant cell defense mechanisms and the most relevant interplay between autophagy and oxidative stress response mechanisms is achieved by Nrf2 signaling. This interplay is an important factor in cellular responses to several physiological and pathophysiological conditions [113]. Moreover, both autophagy and Nrf2 signaling were found to have a protective role against oxidative stress [114,115,116,117,118].

Autophagy and Nrf2 signaling can regulate each other with crucial role of p62-Keap1-Nrf2 positive-feedback loop. Autophagy can activate Nrf2 through competitive interaction between p62 and Keap1. p62 contains a Keap1 interacting region (KIR) motif that allows direct interaction of p62 with Keap1 [114,115,116,117,118]. The consequence is reversal of Keap1 binding to Nrf2, constant nuclear accumulation of non-ubiquitinated Nrf2, and activation of antioxidant genes transcription [119]. It has been documented that post-translational modifications of p62 can have important role in autophagy regulation [120]. The p62 phosphorylation can be induced by free radicals, Additionally, specific phosphorylation at distinct sites is responsible for the realization of different cellular responses. Phosphorylation at serine 351/354 has been shown to increase the p62 binding affinity for Keap1 but the phosphorylation of p62 at serine 349 disrupts protein degradation and autophagy inhibition [121,122].

Furthermore, Nrf2 can have a positive effect on the process of autophagy, whereas nuclear Nrf2 translocation initiates the expression of autophagy and anti-apoptotic genes [123]. In such a way, Nrf2 can promote autophagy and inhibit apoptosis. Intranuclear Nrf2 promotes an overexpression of the *p62* gene via the ARE sequence in the *p62* gene promoter region [114,115,116,117,118,119]. In addition to p62, other autophagy regulators also contain the ARE sequence for Nrf2 binding and the initiation of its expression. Examples include nuclear dot protein 52 (NDP52), Atg4, Atg5, and Atg7 [75,124]. The role of Nrf2 in autophagy regulation is supported by the fact that the Nrf2 activator sulforaphane (SFN) has been shown to play a role in autophagy promotion [125]. The interplay between autophagy and redox signaling was confirmed by several regulatory systems. It has been found that Trehalose, an inducer of mTOR-independent autophagy, can increase the expression of Nrf2 target genes, including p62, leading to a reduction in free radical levels [126]. Tripartite motif (TRIM) 16 protein is a member of the protein family with E3 ligase activities, and was reported to facilitate an increased interaction between p62 and KEAP1, which is associated with Nrf2 activation [127]. The study also demonstrates the role of Nrf2 in protein aggregate formation. Another protein system documenting the interplay between autophagy and redox signaling is NAD-dependent deacetylase Sirtuin 1 (SIRT1). This protein was found to mediate cellular protection via the activation of antioxidant defenses by upregulating and the nuclear deacetylation of Nrf2 [128] and via the regulation (deacetylation) of various autophagy-related proteins [129]. Sestrins (SESN) are stress-inducible proteins that protect cells against a variety of stresses, including DNA damage, hypoxia, oxidative stress, and metabolic stress [130,131]. It was described that SESN2 can initiate the first step of autophagy by regulating AMPK and mTOR kinases [132,133,134]. Importantly, SESN2 was also found to interact with autophagy regulators p62 and ULK1 and to facilitate the recognition and degradation of damaged mitochondria [135]. Moreover, the expression of SESN2 can be induced by an increase in free radicals via the activation of Nrf2 transcription factor. Therefore, SESNs can create a link between the control of oxidative stress response and regulation of autophagy.

**Figure 2:**
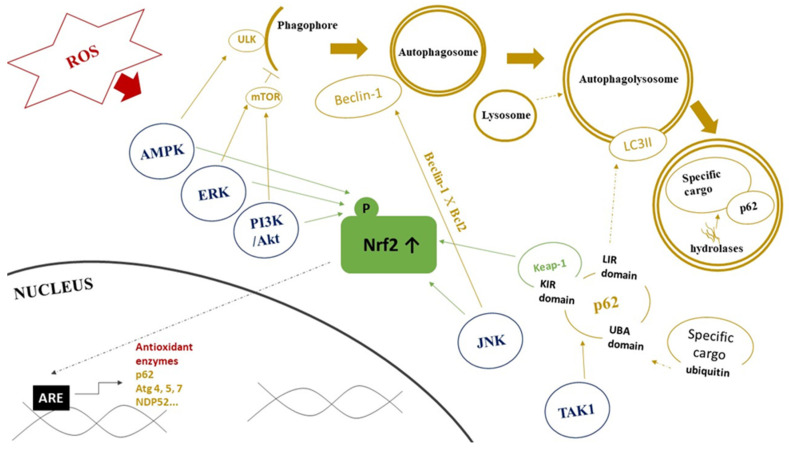
Role of protein kinase pathways in interplay between Nrf2 signaling and autophagy. Abbreviations: ROS—reactive oxygen species, AMPK—AMP-activated protein kinase, ERK—extracellular signal-regulated kinase, PI3K/Akt—phosphatidylinositol 3-kinase/Akt kinase, JNK—c-Jun N-terminal kinase, TAK1—TGF-β-activated kinase 1, ULK—Unc-51-like autophagy activating kinase, mTOR—mammalian target of rapamycin, LC3II—microtubule-associated protein light chain 3-II, UBA domain—ubiquitin-associated domain, KIR domain—KEAP1 interacting region, LIR domain—(LC3)-interacting region, Keap1—kelch-like ECH-associated protein 1, Nrf2—nuclear factor erythroid 2-related factor 2, ARE—antioxidant response element, Atg— AuTophaGy-related proteins. Red color represents components of redox signaling. Blue color represents protein kinases affecting Nrf2 and autophagy. Green color represents components of Nrf2 signaling pathway. Yellow color represents components of autophagy pathway. Dashed line arrows represent the connection/fusion between the two components. Details are provided in the text.

Another mechanisms of both Nrf2 and autophagy regulation involves activities of several protein kinases (Figure 2). Specifically, TGF-β-activated kinase 1 (TAK1), an inflammatory signaling protein kinase, regulates autophagy adaptor p62 to facilitate decreased Keap1 levels, which results in an upregulation of Nrf2 [136]. c-Jun N-terminal kinases (JNKs) and extracellular signal-regulated kinases (ERKs), members of the mitogen-activated protein kinase (MAPK) family, are another upstream effector involved in autophagy regulation in conditions of increased free radicals production [137]. JNK can also regulate autophagy via Bcl-2 phosphorylation. This phosphorylation blocks Bcl-2 interaction with Beclin-1 and its consequence is an inhibition of Beclin-1 function [138]. The activation of the AMPK signaling is another response to oxidative stress. AMPK can stimulate Nrf2-dependent gene expression during oxidative stress through Nrf2 phosphorylation at serine 550 [139]. It was also found to inhibit mTOR and in such way that it directly induces ULK1 activity to enhance autophagy activation [49]. Interestingly, Kosztelnik et al. reported that Nrf2 negatively regulates autophagy during chronic oxidative stress. They attributed this effect to the delayed downregulation of the AMPK expression [140].

Upregulated free radicals can mediate autophagy through several other mechanisms, including the oxidation of autophagy-related proteins such as Atg4, Atg3 or Atg7, all of which contain redox-sensitive cysteine residues [141,142,143], disruption of Bcl-2/Beclin-1 interaction [144], mitochondrial homeostasis alteration, and membrane depolarization, leading to mitophagy [145,146].

## 5. Interplay between Autophagy and Redox Signaling in Cardiovascular Diseases

Oxidative stress and changes in the cellular redox state mediated by an increased production of ROS are associated with a variety of cardiovascular diseases. The changes in redox homeostasis can also regulate autophagy, which may have role in adaptive myocardial responses to disease triggers or maladaptive responses involving cell death induction. In several cardiovascular diseases, a direct interplay between autophagy and redox signaling has been documented [8,29,30,147,148,149,150,151,152,153,154,155]. In Table 1, several examples documenting the interplay between autophagy and Nrf2 in heart failure, ischemic heart disease, and cardiomyopathies are presented. Autophagy disturbances as a consequence of oxidative stress have been identified in many cardiac disease states, including hypertrophy, pressure-overload heart failure, ischemic heart disease, ischemic/reperfusion (I/R) injury, diabetes, and age-related cardiomyopathy [8,93]. In heart diseases, autophagy can play both a protective and detrimental role, depending on the kind of stressor and timing of assessment [156,157,158]. Some studies have revealed a detrimental role of Nrf2 in the progression of cardiovascular diseases such as proteotoxicity associated with aging, myocardial I/R injury, pressure overload (PO), and type 1 diabetes [149,151,153]. The precise mechanisms underlying this contradictory Nrf2 effect on heart function are poorly understood, but recent studies have demonstrated that Nrf2 action could also be mediated through autophagy [151,153]. In a *Drosophila melanogaster* model of cardiac laminopathies, it was observed that increased Nrf2 levels cause autophagy inhibition by mTOR activation, which leads to the inactivation of AMPK. The inhibition of Nrf2 signaling has been shown to be protective, delay ageing, and prolong life span [150].

With regard to myocardial function, it is an important fact that cardiomyocytes contain high numbers of mitochondria. The accumulation of dysfunctional mitochondria can lead to significant alterations in cardiac integrity and function and to pathological cardiac remodeling [154]. The precise elimination of damaged mitochondria through mitophagy is crucial for the function of cardiac cells. An impaired autophagy function was found in cardiomyocytes exposed to chronic oxidative stress, leading to the downregulation of AMPK levels [159]. Furthermore, Nrf2, as a key regulator of antioxidant defenses, has been found to have a protective role in the responses of cardiac cells to pathological conditions associated with oxidative stress [47]. Interestingly, Nrf2 can also promote the autophagic degradation of toxic ubiquitinated proteins by providing a protective effect against cardiac proteotoxicity [30]. The antioxidant defense system formed by p62-Keap1-Nrf2 plays a role here, and the regulatory function of this system has been described in myocardial responses to pathological conditions [147,155]. Free radicals can induce Nrf2-mediated transcription of p62 and autophagy. On the other hand, autophagy can reduce free radical generation and mediate the protection of myocardial cells from apoptosis.

### 5.1. Interplay between Nrf2 Redox Signaling and Autophagy in Ischemic Heart Disease

It has been demonstrated that autophagy can, via Nrf2 signaling activation, improve myocardial infarction (MI) damage [8]. According to another study, myocardial ischemia/reperfusion (I/R) induced mTORC1-mediated p62 phosphorylation at Ser349 [155] (Table 1). This phosphorylation represents a critical step in p62-Keap1 interaction, and its consequence is Nrf2 upregulation. Deficiency in a regulatory subunit of PKA, the inhibitory enzyme for mTORC1, led to the specific inhibition of the autophagic degradation of Keap1 and p. The consequence was a repression of Nrf2 and impairment of the endogenous defense response against oxidative stress [155]. This led to aggravated oxidative stress, cardiomyocyte necrosis, and myocardial ischemia/reperfusion injury. Another study demonstrated that the activation of the p62/Keap1/Nrf2 system with urolithin B (UB), the gut metabolite of polyphenol ellagitannin, can protect cardiomyocytes against I/R injury by decreasing oxidative stress [147]. Moreover, the cardioprotective effects of Salvianolic acid B on acute myocardial infarction in rats were also found to be associated with autophagy promotion and the activation of Nrf2-mediated redox signaling documented by increased levels of superoxide dismutase [160]. The protective effects of Lycium barbarum polysaccharide against ischemia/reperfusion injury in rats and cardiomyocytes via Nrf2 activation through autophagy inhibition were also presented [161]. The interplay between redox signaling and autophagy in ischemic heart disease is supported also by findings that resveratrol provides cardioprotection against I/R injury via redox signaling activation and autophagy induction [162].

### 5.2. Interplay between Nrf2 Redox Signaling and Autophagy in Cardiomyopathies

The development of cardiovascular diseases as a consequence of autophagy and redox signaling disturbances has been documented in several studies. It has been found that the cardiac deletion of Atg5 or Pink1 in mice resulted in age-related cardiomyopathies that were associated with mitochondrial dysfunction and oxidative stress [56,163]. Insufficient autophagy was also found to be associated with Nrf2-related exaggeration of the progression of diabetic cardiomyopathy in mice [149].

Cardiomyopathy induced by the cytostatic agent doxorubicin (Dox) also suggests a relation between Nrf2 and autophagy. It has been found that Dox-induced increases in free radical levels leads to an alteration of the autophagy function, disruption of mitochondria, and subsequent cardiomyocytes damage [29] (Table 1). Furthermore, Nrf2 activation was found to mediate the reversal of Dox-induced negative effects and protect the heart against failure. Dysregulation of autophagy and redox signaling plays an important role in DOX-induced cardiotoxicity. This is supported by the finding that combined treatment with carvedilol (CAR) and carnosic acid (CAA) attenuates doxorubicin-induced cardiotoxicity and the protective effects have been associated with the suppression of excessive autophagy and oxidative stress [164]. An improvement in cardiac performance and redox homeostasis by CAR and CAA included the augmenting of anti-oxidative enzymes expression and activities. Further therapeutic approaches based on the modulation of both the Nrf2 signaling pathway and autophagy are presented [161,165,166]. A model of doxorubicin-induced cardiotoxicity in mice presented data showing the cardioprotective potential of a natural quinone β-LAPachone via a modulation of both the AMPK-Nrf2 and AMPK-mTOR signaling pathways [165]. Luo et al. found that antioxidant allopurinol (ALP) maintains inner redox homeostasis and attenuates diabetic cardiomyopathy in rats via the restoration of the Nrf2/p62 signaling pathway and through normalizing disordered autophagy [166].

The crosstalk between Nrf2 and autophagy as an effect of Dox has also been described by Hou et al. [148] (Table 1). According to this study, TRIM21 E3 ubiquitin ligase interacts with p62 and negatively regulates the antioxidant p62/Keap1/Nrf2 pathway. Another possible protecting agent is sestrin 2 (SESN2), which has been shown to activate Parkin-mediated mitophagy and to improve mitochondrial function after exposure to doxorubicin [167]. Mitophagy in failing heart can also be affected by AMPK, which phosphorylates PINK-1 at Serine 495, making it essential for effective mitophagy and heart failure prevention [154].

### 5.3. Interplay between Nrf2 Redox Signaling and Autophagy in Heart Failure

Qin et al. described that Nrf2 plays a protective role in hearts exposed to pressure overload (PO) when the autophagy function is sufficient, but an alteration in autophagy caused negative Nrf2-mediated effect on PO hearts in their study [151] (Table 1). This detrimental effect was caused by Nrf2 nuclear accumulation and the concomitant transcription of angiotensinogen, a factor involved in pathological cardiomyocytes remodeling. The inactivation of the Jak2/Fyn signaling pathway is probably also involved. This pathway is responsible for nuclear export of the phosphorylated Nrf2, inactivation of Nrf2-driven gene expression, and Nrf2 degradation in cytoplasm [168]. Wu et al. performed another study using a mouse model of PO-induced cardiomyopathy and heart failure, where autophagy and Nrf2 activity were genetically inhibited [153]. They found that the upregulation of myocardial expression of angiotensinogen is caused by Nrf2 signaling. They concluded that this effect is most likely caused by the inactivation of ERK kinase during autophagy inhibition and that autophagy activation may reverse these effects. These results confirmed the key role of autophagy in cardiac homeostasis and showed that the activation of autophagy is essential for Nrf2-mediated cardioprotection.

## 6. Conclusions

Oxidative stress is responsible for many injuries in organisms and can lead to the development of several diseases. It plays a very important role in cardiovascular diseases. Cells have developed effective mechanisms to avoid these injuries and to maintain normal cellular homeostasis. The Nrf2 signaling pathway, the key regulator of the cellular redox state, and autophagy, the lysosome-self-repair system, play an important role here.

In summary, several studies suggest that Nrf2 and autophagy can, in tandem, suppress the development of cardiovascular diseases, particularly through the p62/Keap1/Nrf2 feedback loop and by reducing free radicals levels. Interactions between the actions of some protein kinase signaling pathways and both Nrf2 and autophagy in relation to cardioprotection have also been identified. Current information indicates that the modulation of autophagy through Nrf2 could present a promising strategy for the treatment of cardiovascular diseases, in which oxidative stress is an important partner. However, it is still important to develop a better understanding of the interplay between Nrf2 signaling and autophagy and their common role in mechanisms of cardioprotection.

## Figures and Tables

**Table 1:** Interplay between autophagy and Nrf2 in cardiovascular diseases.

Disease	Model	Sample	Autophagy	Autophagy-Redox Signaling	Reference
Heart failure	TAC operation	Mice; Rat cardiac myocytes (H9c2)	Enhanced autophagosome formation and autophagic flux	Nrf2 increases autophagy-mediated clearance of ubiquitinated protein aggregates in cardiomyocytes	[30]
	TAC operation	Mice	-	When autophagy is intact, Nrf2 is required for cardiac remodeling. When autophagy is impaired, Nrf2 nuclear export is decreased and Nrf2-driven angiotensinogen transcription is increased, which can lead to cardiac dysfunction	[151,153]
	TAC operation; patients with heart transplantation	Mice, human	Defective mitophagy	AMPK improves mitophagy via PINK-1 phosphorylation and decreases ROS formation	[154]
Ischemic heart disease	LAD ligation	Mice	-	Autophagy increases Nrf2 signaling activation, which leads to MI damage improvement	[8]
	I/R (LAD ligation)	Mice; NRCMs	Impaired mTORC-p62-Keap1-Nrf2 antioxidant defense system	Impaired mTORC-p62-Keap1-Nrf2 antioxidant defense system	[155]
	I/R (LAD ligation) Urolithin B treatment	Rats	Urolithin B decreases autophagy by Akt/mTOR/ULK1 pathway	Urolithin B increases p62/Keap1/Nrf2 signaling pathway activation	[147]
Doxorubicin-induced cardiomyopathy	Doxorubicin treatment	Mice	Defective autophagy	P62-Keap1-Nrf2 activation leads to the positive regulation of oxidative stress and autophagy	[29,148]
Cardiac laminopathy	Lamin C mutant	*Drosophila melanogaster*	Increased autophagy genes expression	Increased Nrf2 levels lead to autophagy inhibition by mTOR activation	[150]
Diabetic cardiomyopathy	Type 1 diabetes	Mice	Defective autophagy	Autophagy inhibition leads to increased Nrf2 levels and thus to the progression of diabetic cardiomyopathy	[149]

## Data Availability

Not applicable. No data were generated or analyzed in this work.
